# Natural variation in OsbHLH091 fine-tunes rice floret opening time in a JAZ-mediated dance

**DOI:** 10.1093/plcell/koag259

**Published:** 2026-09-01

**Authors:** Ju-Chen Chia

**Affiliations:** Assistant Features Editor, The Plant Cell, American Society of Plant Biologists; Plant Biology Section, School of Integrative Plant Science, Cornell University, Ithaca, NY 14853, United States

Crosses between rice cultivars can produce hybrids with superior agronomic traits compared with their parental cultivars (Huang et al. 2015). However, hybridization between *indica* and *japonica* is often hindered by differences in their diurnal floret opening time (DFOT). In general, *indica* cultivars exhibit an earlier DFOT, typically between mid-morning to noon, whereas *japonica* cultivars generally open their florets 2 hours later. Understanding the mechanisms controlling DFOT is therefore important for improving hybrid rice production.

Rice floret opening and closure follow a diurnal rhythm and are driven by dynamic changes in lodicules, a pair of small organs at the base of the floret that are analogous to petals in dicots (Yoshida 2012; Yan et al. 2022). Lodicules swell upon hydration to promote opening and shrink as they lose water to trigger closure. Previous studies have shown that jasmonic acid (JA) and the master JA-responsive transcription factor OsMYC2 regulate lodicule cell wall remodeling and expansion, thereby promoting floret opening (Liu et al. 2017; Zhu et al. 2024). JASMONATE ZIM-DOMAIN (JAZ) proteins repress JA responses by sequestering JA-inducible transcription factors and may thus contribute to DFOT regulation in rice (Pauwels and Goossens 2011; Wang et al. 2024).

Despite the well-established role of JA in floret opening, the molecular mechanisms underlying this pathway have remained largely unknown. To address this gap, Tao Song and colleagues (Song et al. 2026) performed RNA sequencing of lodicules isolated from rice florets before, during, and after anthesis and identified a novel bHLH transcription factor gene, *OsbHLH091,* which showed a sharp expression peak at anthesis and was specifically expressed in lodicules. *OsbHLH091* knockout mutants in 2 different *japonica* cultivars exhibited cleistogamy, in which florets remain closed during anthesis and undergo self-pollination. Both fresh weight and volume of the *bhlh091* lodicules were reduced compared with those of wild-type lodicules. The significantly reduced lodicule size eventually resulted in insufficient force to drive floret opening. Other pollination-related phenotypes, including filament elongation, pollen viability, and anther dehiscence, remained unaffected in the mutants, suggesting that OsbHLH091 is specifically involved in cell wall remodeling in lodicules during anthesis.

To identify the downstream targets of OsbHLH091, the authors compared the transcriptomes of lodicules from the *bhlh091* mutant and wild-type plants. The OsbHLH091-dependent regulon appeared to function independently of the canonical OsMYC2 pathway, suggesting 2 distinct mechanisms regulating floret opening. Protein-DNA interaction analyses showed that OsbHLH091 directly binds the promoters of several cell wall–modifying genes, including *OsEXPA1*, *OsEXPB4*, and *OsCESF6*. Overexpression of *OsEXPB4* in the *bhlh091* mutant rescued its delayed DFOT phenotype, suggesting that OsbHLH091 regulates floret opening through *OsEXPB4* and likely other cell wall–modifying components. Conversely, *OsbHLH091* overexpression increased lodicule size, enhanced the expression of *OsEXPA1*, *OsEXPB4*, and *OsCESF6*, and promoted earlier DFOT, further supporting the role of OsbHLH091 in lodicule expansion and floret opening.

The authors then asked whether OsbHLH091 is involved in the JA signaling pathway. Their results suggested that OsbHLH091 is unlikely to be involved in JA biosynthesis but instead might act in the JA signaling pathway. The authors observed that OsbHLH091 physically interacted with 6 JAZ proteins (repressors of JA-induced transcription), with OsJAZ11 showing the strongest interaction. When *OsJAZ11* was co-expressed with *OsbHLH091* in plant tissues, OsbHLH091 failed to activate the promoters of *OsEXPB4*, *OsEXPA1*, and *OsCESF6*, whereas JA treatment relieved this repression, presumably through OsJAZ11 degradation.

Interestingly, the authors found that natural variation in OsbHLH091 contributes to differences in DFOT between *indica* and *japonica* subspecies (see Fig. 1). The OsbHLH091 protein encoded by *japonica* alleles interacts more strongly with OsJAZ11, resulting in stronger repression of OsbHLH091 and delayed floret opening. Specifically, residue 312 of OsbHLH091, which is arginine in the *japonica* haplotype and proline in the *indica* haplotype, affects the binding affinity between OsJAZ11 and OsbHLH091. Mass spectrometric analysis revealed that this polymorphism alters the phosphorylation status of adjacent serine residues. Point mutations at these phosphorylation sites in turn affected the OsbHLH091–OsJAZ11 interaction. However, the kinase responsible for this phosphorylation remains to be identified.

**Figure 1:**
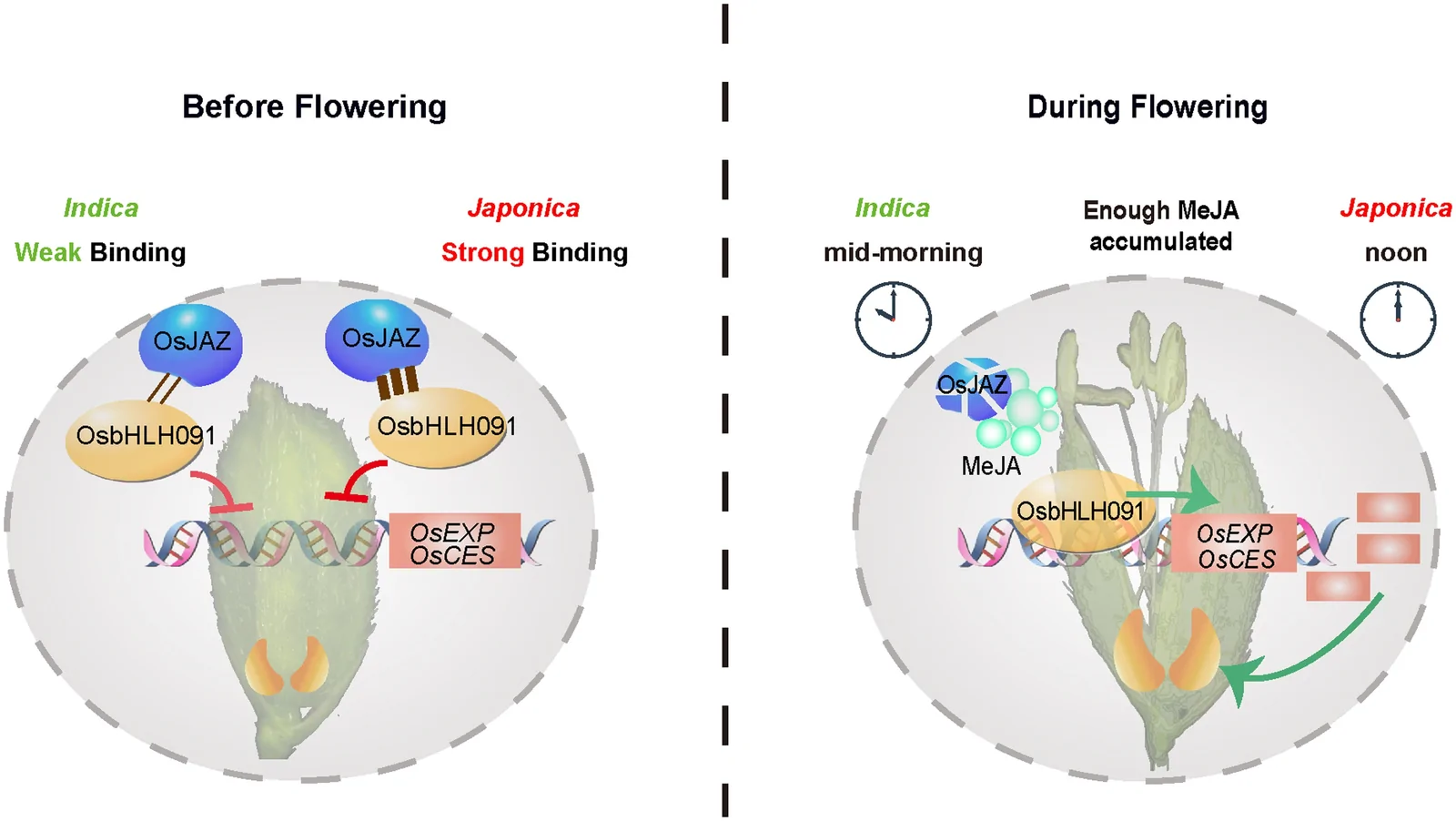
Working model of the JA-OsJAZ-OsbHLH091 pathway in rice lodicules. OsJAZ interacts with OsbHLH091 to repress the expression of *OsEXP* and *OsCES* genes, whereas methyl-jasmonate (MeJA) promotes OsJAZ degradation, releasing OsbHLH091 from repression. The weaker OsJAZ11-OsbHLH091 interaction in *indica* allows earlier activation of *OsEXP* and *OsCES* by OsbHLH091, promoting floret opening, whereas the stronger interaction in *japonica* delays OsbHLH091 activation and subsequent floret opening. Adapted from Song et al. (2026), Fig. 9.

In summary, Song et al. identified a novel JA-OsJAZ11-OsbHLH091 pathway in lodicules that controls floret opening by regulating cell wall remodeling (Fig. 1). These findings demonstrate how a single nucleotide polymorphism (SNP) can alter a protein–protein interaction to fine-tune an agronomic trait. Identifying the specific kinase responsible for OsbHLH091 phosphorylation will provide further insights into the molecular mechanisms regulating floret opening through the JA-OsJAZ11-OsbHLH091 pathway. More importantly, the lodicule-specific expression and minimal effects on other developmental traits make *OsbHLH091* a promising candidate for genetic engineering compared with the broadly acting JA regulator *OsMYC2*.

## Recent related articles in *The Plant Cell*

- Zhao et al. (2026) identified a MYB coiled-coil-type transcription factor, APL, as a key regulator of flowering time in maize.
- Li et al. (2026) identified the distinct functions of 3 jasmonate-amido synthetases in rice that contribute to floral organ formation.
- Chen et al. (2025) identified a JAZ-Polycomb repressive complex (PRC) mechanism that inhibits MYB26 activity, thereby repressing genes required for secondary cell wall formation and anther development.
- Zhang et al. (2025) identified a JA signaling pathway regulating seed size.
- Eng et al. (2026) showed that CELLULOSE SYNTHASE 7 (CESA7) and cortical microtubules coordinately regulate secondary cell wall patterning in the explosive fruits of *Cardamine hirsuta*.

## Data Availability

No new data were generated or analyzed in support of this research.
